# American Indian and Alaska Native recruitment strategies for health-related randomized controlled trials: A scoping review

**DOI:** 10.1371/journal.pone.0302562

**Published:** 2024-04-30

**Authors:** Nicole Redvers, Sarah Larson, Olivia Rajpathy, Devon Olson

**Affiliations:** 1 Schulich School of Medicine & Dentistry, Western University, London, ON, Canada; 2 Department of Indigenous Health, School of Medicine & Health Sciences, University of North Dakota, Grand Forks, ND, United States of America; 3 Department of Population Health, School of Medicine & Health Sciences, University of North Dakota, Grand Forks, ND, United States of America; 4 Library Resources, School of Medicine & Health Sciences, University of North Dakota, Grand Forks, ND, United States of America; UCSF: University of California San Francisco, UNITED STATES

## Abstract

**Background:**

Significant health disparities exist among American Indian and Alaska Natives (AI/ANs), yet AI/ANs are substantially underrepresented within health-related research, including randomized controlled trials (RCTs). Although research has previously charted representation inequities, there is however a gap in the literature documenting best practice for recruitment techniques of AI/ANs into RCTs. Therefore, the aim of this review was to systematically gather and analyze the published literature to identify common strategies for AI/AN participant recruitment for RCTs in the US.

**Methods:**

A scoping review methodology was engaged with a systematic search operationalized within relevant databases to February 19, 2022, with an additional updated search being carried out up until January 1, 2023: PubMed, Embase, Web of Science, PsycINFO, CINAHL, and Google Scholar. A two-stage article review process was engaged with double reviewers using Covidence review software. Content analysis was then carried out within the included articles by two reviewers using NVivo software to identify common categories within the data on the topic area.

**Results:**

Our review identified forty-one relevant articles with the main categories of recruitment strategies being: 1) recruitment methods for AI/ANs into RCTs (passive advertising recruitment approaches, individual-level recruitment approaches, relational methods of recruitment); 2) recruitment personnel used within RCTs; and, 3) relevant recruitment setting. The majority of the included studies used a culturally relevant intervention, as well as a community-involved approach to operationalizing the research.

**Conclusion:**

Increasing AI/AN representation in RCTs is essential for generating evidence-based interventions that effectively address health disparities and improve health outcomes. Researchers and funding agencies should prioritize the engagement, inclusion, and leadership of AI/AN communities throughout the RCT research process. This includes early community involvement in study design, implementation of culturally tailored recruitment strategies, and dissemination of research findings in formats accessible to AI/AN communities.

## Introduction

Significant health disparities exist among many minority populations in the United States (US), including higher mortality [1] and cancer rates [2] among American Indians and Alaska Natives (AI/AN) [3]. AI/ANs also face a disproportionate burden of morbidity, including diabetes, alcohol use, and tuberculosis compared to non-Hispanic Whites [4]. Despite this, AI/ANs are substantially less represented within health-related research. Unfortunately AI/AN research that has been carried out has historically been wrought with ethical atrocities and violations of human rights [5] that has negatively impacted community trust with researchers and academic institutions broadly. Boarding schools, forced sterilization, and the misuse of blood samples are a few examples, among others, that have contributed to community trauma and a lack of trustworthiness between AI/AN communities and medical and research institutions [6–8]. To increase beneficial research within AI/AN communities, it has been stated that acknowledging the wrong that has occurred while building up community-based research methods may be a way forward towards establishing better relations [9]. Special consideration is therefore needed when seeking participation from AI/AN populations within research processes [10].

Randomized controlled trials (RCTs) are often considered the gold standard for medical-related research, despite some noted limitations. RCTs attempt to standardize the research process by providing a mechanism for more easily controlling for myriad variables, ensuring validity, as well as reducing the chances of biased results to establish the effectiveness of a treatment or intervention [11]. Appropriate participant representation within RCTs, however, impacts the generalizability of study findings, and therefore health outcomes of communities and populations included in the research [12]. Despite the United States Congress mandating an increase of representation in research of minorities to reduce health disparities in 1993 [13,14], AI/ANs continue to be underrepresented in RCTs as well as other types of research. Recent reviews have documented underrepresentation for AI/ANs in many health-related areas of clinical studies, including cancer [15], Pfizer drug trials [16], Glaucoma [17], vaccines [18], Alzheimer’s [19], depression [20], and alcohol use disorder [21]. Overall, although participation has increased in recent years [22], as of 2017 there was still only 1% of AI/ANs enrolled in NIH clinical studies [23,24]. To reduce health disparities and improve health outcomes it is imperative that research, including RCTs, not only include, but also centre AI/AN individuals and communities.

Participation in RCTs is impacted by many factors, including how a research project is undertaken. For example, community-based participatory research (CBPR) has been shown to have positive effects on recruitment efforts [25,26]. Increasing representation does, however, also require appropriate population-level recruitment methods. Although research has previously charted representation inequities, there is, however, a gap in the literature documenting best practice for recruitment techniques of AI/ANs into RCTs. A review published in 2015 charted the barriers and facilitators for Indigenous participation in randomized controlled trials at the international level [26] and found relationship building and targeted recruitment being potentially positive strategies. This review was broad and did not focus specifically on the unique context of AI/ANs within the US. Therefore, there was a need to gain a clearer understanding of effective RCT recruitment strategies for research with AI/AN populations specifically. Having a better understanding of the RCT recruitment strategies for AI/ANs may help to increase research participation, improve community trust in future research endeavours, and support the creation of research protocols that have a higher chance of increasing the body of evidence for interventions to address AI/AN health disparities.

With this, we developed a scoping review protocol with the aim to systematically gather and analyse the published literature to identify common strategies for AI/AN participant recruitment for RCTs in the US. A scoping review framework was chosen due to its flexibility to chart the data iteratively while being responsive to identified gaps given the lack of currently focused discourse on the topic as it pertains to the US context. Our overarching goal was to increase understanding while additionally amplifying potentially effective strategies for recruitment of AI/AN into RCTs. Given our overarching goal and aim, our review didn’t seek to determine the validity of study outcomes themselves, but to gather the available evidence related to how recruitment has been conducted successfully in AI/AN communities. We specifically wanted to identify the participant recruitment strategies used to engage AI/ANs in health-related randomized controlled trials in the US. Therefore, the objectives of this scoping review were to: 1) chart the current RCT literature inclusive of AI/AN participants and the recruitment strategies used, 2) clarify common recruitment strategies as referenced by the included articles, and 3) reflect on identifiable literature gaps on AI/AN recruitment for RCTs.

## Methods

The methodology used for this scoping review followed the outline and framework developed by Arksey and O’Malley [27], which was further refined by Peters et al [28]. The PRISMA-ScR checklist [29] was engaged to ensure appropriate reporting standards for scoping reviews. The study protocol was registered in the OSF (Open Science Framework) Registries database prior to creation of data [30].

### Procedures, search terms, and eligibility criteria

A medical librarian (DO) with experience in literature search strategies was consulted to support the development of the systematic search strategy (see *S1 Appendix file* for master search phrases). The following electronic databases were searched up to February 19, 2022, with an additional updated search being carried out up to January 1, 2023: PubMed, Embase (Elsevier), Web of Science (Clarivate), PsycINFO (EBSCO), and CINAHL (EBSCO). Google Scholar was searched by reviewing the first two pages, then screening the next two pages until no relevant articles were found (with relevance framed by our inclusion criteria as per further below). Additional manual searches were carried out to identify relevant articles in: iPortal (Indigenous Studies Portal), the Native Health Database, and the International Journal of Indigenous Health database. The reference lists of key articles were searched to further identify relevant articles that were not found during the initial search steps. Key experts were additionally contacted to enquire about their knowledge of key studies in the field.

Studies were included if they were health-related RCTs conducted in the US with adults, including mental and behavioural health studies; however, we did not include studies with group-level randomization. Studies were only included if they explicitly stated recruitment strategies for AI/AN participation in the study. There was also no restriction on the date of publication, with only English-language articles being considered for inclusion. Relevant articles from the systematic search were subsequently exported into the Covidence review software (v2721 a9510157) to facilitate the study selection process.

### Article screening

A two-stage article review process was engaged using Covidence review software. The first step included a title and abstract screen that was carried out by two independent reviewers (SL, OR), with a third reviewer (NR) brought in to discuss and resolve any discrepancies through discussion. The second step included full-text screening with again 100% double-screening by two independent reviewers (SL, OR). A third reviewer (NR) was brought in to resolve any discrepancies in the full-text review through discussion.

### Data characterization and analysis

Data extraction from the included articles was carried out by one reviewer (SL) with a second reviewer (OR) cross-checking all the articles for accuracy. A third reviewer (NR) was brought in to settle discrepancies through discussion. Data was charted in Excel 365 under the following categories: general article information, recruitment method(s), number of AI/AN participants, rural or urban study population (if known), geographic location of study, and specific Tribal population (if specified). Content analysis as described by Elo and Kyngäs [31] was then carried out within the included articles by two reviewers using NVivo software (Release 1.3) with relevant codes organized into common categories within the data on the topic area. We did not carry out quality appraisal on the included articles, as our overarching goal and aim of the review, as noted previously, didn’t seek to determine the validity of study outcomes themselves, but to instead gather the available evidence related to how recruitment has been conducted successfully in AI/AN communities.

## Results

**Fig 1:**
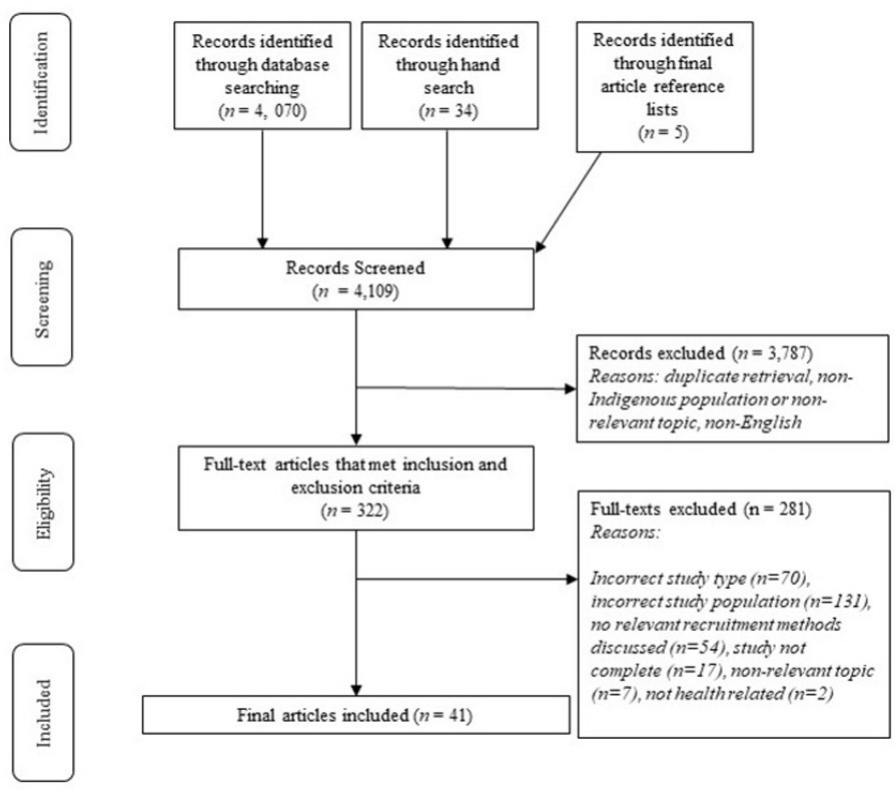
Adapted PRISMA flow chart.

There were 4109 articles identified through the systematic search (see *Fig 1*). A total of 41 studies were ultimately included in the review. Seven of the included articles had more than one publication associated with the study [32–38]. For example, articles that were published with study protocols before the study was completed were designated as “DYADs” to the final study publication (see *S2 Appendix*) [39–45]. Therefore, there were 48 articles actually analysed, but only 41 unique studies included in the final list for the purposes of this review. The reason for including the DYADs for review was that many studies referenced their study protocols for the recruitment methods used, which was important to capture without counting them as independent papers. The most common research topic areas of the included articles included: physical health, substance use, mental health, cancer screening, smoking, vaccination, sexual health, and behavioural health. The studies were conducted within various regions in the US, including in both urban and rural contexts, with sixteen of the studies being carried out in either Alaska or the Pacific Northwest. Specific Tribal groups were only explicitly identified in ten of the included studies, and the number of AI/AN study participants varied widely from 25 to 5,363 participants in a respective study (see *S2 Appendix* for full data extraction on all included articles).

Our content analysis identified three overarching categories surrounding recruitment strategies for AI/AN participants into RCTs, including: recruitment methods for AI/ANs into RCTs, recruitment personnel used within RCTs, and relevant recruitment settings for RCTs. These categories are detailed further below. Notably, 25 of the 41 included studies used a culturally adapted intervention that incorporated and included some aspect of the community’s cultural practices. Sixteen of the 34 studies stated that they had used a Community Based Participatory Research (CBPR) approach or a Community Advisory Board (CAP); however, only four of the 34 studies stated they had gathered community input on recruitment or recruitment strategies explicitly.

### Recruitment methods for AI/ANs into RCTs

Within the recruitment methods for AI/ANs into RCTs category, we identified three subcategories, including: 1) passive advertising recruitment approaches, 2) individual-level recruitment approaches, and 3) relational recruitment methods. It is important to note that the included articles often reported using several recruitment methods together with passive advertisement and relational recruitment methods used most often within the included studies.

*Passive advertising recruitment approaches* as a recruitment method was used in 23 of the 41 included studies with advertisements placed around the community within clinics and newspapers, as well as posters placed around the communities of interest [42,46,47]. Digital advertisements specifically were used on platforms such as social media, television, radio, or other online spaces in 18 of the included studies, with community radio advertising being the most commonly used [48–51]. Researchers were very intentional about selecting platforms that were particularly relevant with special effort made “to recruit AI/AN participants using Web sites and media associated with Tribal and AI/AN organizations” [52].

*Individual-level recruitment approaches* included methods such as contact via phone in six studies, and by letter or mail in eleven studies. Potential participants were identified through medical records in nine of the 34 included studies, with study staff then being able to reach out for more specific recruitment after confirming eligibility [45,47,53]. These types of recruitment methods did not necessarily involve any previous relationship with potential participants; however, they often required a personalized initial contact. Additionally, several studies sent out information through the mailing system to identified eligible participants, and followed up with a phone call to gauge interest [41,45,54].

*Relational recruitment methods* included personal referrals, word of mouth, and home visiting as ways to connect with potential participants. The majority of the included articles included this form of recruitment within RCTs with AI/ANs *(n* = 21). Referrals were made by providers, case managers, and also family and friends depending on the study [40,51,55]. Although logistically it can be convenient to establish referral methods within a healthcare system to gain study participants, word of mouth was noted to be very powerful to engage a community. One study in particular noted, that “[it] is our experience that word of mouth recruitment (i.e., referrals from current participants) was the most effective strategy for recruitment” [43].

### Recruitment personnel used within RCTs

The kinds of personnel used for participant recruitment within AI/AN specific RCTs was identified as being an important part of recruitment in some of the included studies. Individuals involved in the recruitment of AI/AN participants appeared to have a very substantial role to play as well as a large impact on gaining the trust of potential participants. Six of the included studies reported using recruitment personnel [47,56–60], with two studies noting specifically that they involved AI/AN community members, an “AI Tribal liaison and AI clinical coordinator” [56] and/or “community outreach trainers” [60]. Other studies reported the use of study staff for recruitment activities, naming them generally as “study personnel” or “research staff” without further specifying their level of connection with the community of participants [47,57].

### Relevant recruitment settings for RCTs

It was evident that the recruitment setting was an important variable across many of the included studies to most effectively carry out targeted AI/AN recruitment for the best results and highest engagement. The recruitment settings that were identified within the studies included Tribal and Urban Indian Health (UIH) clinics, community outreach events, Native health fairs, and within online environments [35,43,51,61]. Thirteen of the included studies reported recruiting participants at Tribal or Urban Indian Health (UIH) clinics or facilities, indicating the potential benefit of leveraging existing AI/AN healthcare settings for participant recruitment. Utilizing community outreach events was also a popular strategy reported in thirteen studies. Community outreach events included “community gatherings and Tribal meetings” [46]. For example, one study directly mentioned participating actively in “community events (e.g., bingo, cultural events)” for purposes of AI/AN recruitment [50]. Additionally, AI/AN study recruitment being carried out at Native health fairs was specifically mentioned in seven of the included studies. Although overlapping with community outreach events, given the frequency of Native health fairs being specifically noted as a recruitment technique being utilized, it is reported separately. Online recruitment settings were mentioned in eight of the included studies, highlighting the potential of virtual platforms for reaching AI/AN populations. Social media was usually cited generally; however, Facebook as a platform was mentioned specifically five times [43,51]. One nationwide, online study, for example, not only used Facebook ads, but also “a survey sampling company” [51].

## Discussion

Our review identified forty-one relevant articles with the main categories of recruitment strategies being: 1) Recruitment methods for AI/ANs into RCTs, 2) Recruitment personnel used within RCTs, and 3) Relevant recruitment settings for RCTs. It was apparent throughout the included articles that the participation of AI/AN populations in clinical trials towards completion was dependent upon the success of community-level recruitment activities. Many AI/AN communities require unique settings for recruitment that researchers should be attuned to, such as Native health fairs and other community events. It is important to highlight that researchers across the included studies often used several recruitment methods in tandem, especially passive advertising approaches. Additionally, although more modern forms of technology have an increasingly widespread impact in AI/AN communities for research communications and potential recruitment, community radio was still found to be an effective tool for reaching AI/AN community members.

Similar recruitment method findings have been identified at the international level outside of the United States when it pertains to Indigenous participants within RCTs. For example, community engagement and community involvement within the RCT, as well as the importance of recruitment in relevant settings have been reported elsewhere [26]. Given this, there may be some overlap with regards to successful recruitment practices across wider Indigenous Nations that could be looked to for examination within local contexts. Regardless, it is important to keep in mind that there is substantial heterogeneity across AI/AN contexts that need to be front of mind when co-designing appropriate recruitment methods for RCTs. The effectiveness of RCT recruitment strategies with other minority populations therefore may also not be directly comparable to AI/AN populations and care is needed to ensure the combination of recruitment approaches are nuanced to the AI/AN context. Overall, effective strategies catered and streamlined towards the relevant population are necessary for effective recruitment success [62].

It must be highlighted that there was an overall lack of detailed reporting of the respective recruitment methods in the included articles with some only specifying a listing of the methods used. Given this, it is difficult to conclude the overall efficacy of certain individual methods; however, authors did occasionally make suggestions and noted positive recruitment facilitators such as community-based involvement (i.e., efforts made to platform the communities’ perspectives in the design, facilitation, and operationalization of the intervention), specified incentives (e.g., mainly gift cards, occasionally other small gifts), and hiring local AI/AN study staff. Given the lack of detail reported on the specifics of the recruitment methods used, it is also possible that more detailed recruitment methods were used but were just not reported on. Studies have indicated the general lack and need for better reporting of recruitment methods in RCTs [63]. More comprehensive reporting of recruitment methods as well as strategies in RCTs could contribute to a better understanding of the nuances of successful recruitment strategies for AI/AN populations as well as provide an opportunity to tailor recruitment methods and strategies to specific minority populations or subgroups in general.

As noted previously, including the AI/AN community involved in the research in the overall research planning including the recruitment process (i.e., community-based involvement) appeared to be important not only in increasing recruitment and retention within the RCTs, but also to improve trust. Community-based engagement or involvement has been found to be an effective approach for improving recruitment in various study designs due to its versatility, acceptability, relevance, and overall effectiveness in various contexts of research [64]. ‘Community’ plays a huge role in AI/AN research involvement so community-informed approaches for this population can be very relevant and respectful, and embody a sensitive approach [65]. Additionally, AI/AN communities often have tight-knit communities where relational referrals can be effective; however, if negative or harmful experiences occur (as has been the case with research in AI/AN communities for decades), then word can also spread fast in communities, limiting the effectiveness of overall recruitment strategies. Addressing trust barriers requires ongoing efforts to build back trust by establishing partnerships, and demonstrating respect for AI/AN cultures and communities. Overall, community involvement and participation allows for shared desire for outcomes, shared responsibilities, and facilitating collaboration [65].

Lastly, in some of the included articles, authors explicitly discussed challenges and issues that arose with participant recruitment within their RCT. Challenges included having a limited budget for recruitment, as well as being able to work with the preexisting barriers that exist within AI/AN communities [66]. For example, some barriers included the lack of available transportation to the study sites (i.e., geographic barriers), participants not having the time to participate, and cultural barriers that may affect views on certain treatments or interventions [67,68]. Five studies, for example, were not able to recruit their initial target number, and some adjusted the eligibility criteria or recruitment strategy to obtain more participants [43,69]. Another study reported that they brought their recruitment issues to the “Community Advisory Board,” which culminated in broadening “two inclusion/exclusion criteria, decreas[ing] the number of sessions, and allow[ing] participants to continue the program to enhance feasibility and recruitment into the study” [70]. Regardless, to improve access to RCT participation for AI/AN communities, better efforts need to be made to ensure more flexible study protocols and supports that align with the realities faced in AI/AN communities.

### Limitations

One noted limitation of this review is that the overall number of RCTs specifically targeting AI/AN populations in the US remains very limited. Given this, there is a general lack of research pertaining to effective strategies for RCTs in the published literature for this population. This means that the ability to generalize our findings to all contexts would be limited. Despite this, given the consistency of our findings across varied geographic, urban and rural, and Tribal contexts we feel there are some lessons learned that can be considered in other contexts. This lack of available RCT studies in AI/AN populations also highlights the need for more focused research on addressing health disparities and improving health outcomes among AI/AN communities. Our review also does not provide any outcome-related information as our goal was to get a general idea of the spread of recruitment strategies across the included studies. Despite this, we assumed that if a study was successful enough to be reporting results in a paper, that there had to be some level of success with recruitment of AI/AN participants, and there were lessons to be learned from their approach. Regardless, as many studies often briefly reported their recruitment strategies, there is a substantial need for more research evaluating specific recruitment strategies to determine their efficacy in increasing AI/AN research participation in specified contexts.

## Conclusion

The findings of this scoping review have important implications for RCT research and practice within AI/AN communities. Increasing AI/AN representation in RCTs is essential for generating evidence-based interventions that effectively address health disparities and improve health outcomes. Researchers and funding agencies should prioritize the engagement, inclusion, and leadership of AI/AN communities throughout the RCT research process. This includes early community involvement in study design, implementation of culturally tailored recruitment strategies, and dissemination of research findings in formats accessible to AI/AN communities. Furthermore, reporting standards should be improved to ensure better documentation and reporting of AI/AN-specific representation in RCT research, including the unique reporting needs for recruitment (e.g., level of community partnership specified). Transparent reporting allows for more easily monitoring progress and accountability in addressing the underrepresentation of AI/AN populations in RCT research.

## Supporting information

S1 Appendix Master search phrases. (PDF)

S2 Appendix Full data extraction table. (XLSX)

S1 File (PDF)
