# Supplementary material for: American Indian and Alaska Native recruitment strategies for health-related randomized controlled trials: A scoping review
Source: PLoS One. 2024 Apr 30;19(4):e0302562. doi: 10.1371/journal.pone.0302562 (PMC11060564; doi:10.1371/journal.pone.0302562)
Supplement: S1 Appendix — (PDF) [file pone.0302562.s001.pdf]

Additional file 1

Master search phrases for PubMed, Embase, Web of science, Psych Info, Google Scholar, CINAHL iPortal Indigenous Studies Portal, Native Health Database, International Journal of International Journal of Indigenous Health Database

Search Strategy:

**Database**	**Search Phrase**<br>**Randomized Control Trial and American Indian/Alaska Native search strings always combined with "AND" and searched as a single string in databases*	
	**Randomized Control Trial**	**American Indian (Alaska Native)**
Synonyms	("Randomized Control Trial" OR "Randomized controlled trial" OR RCT)	("American Indian" OR "Alaska Native" OR "Native American" OR Indigenous OR tribe OR tribal OR reservation OR "American Indian/Alaska Native" OR AI/AN OR AIAN OR "Indian Country" OR "urban Indian")
PubMed*	("Randomized Controlled Trials as Topic"[Mesh] OR "Randomized Controlled Trial" [Publication Type] OR "Randomized Control Trial" OR "Randomized controlled trial" OR RCT)	("Alaskan Natives"[Mesh] OR "Indians, North American"[Mesh] OR "American Indians or Alaska Natives"[Mesh] OR "American Indian" OR "Alaska Native" OR "Native American" OR Indigenous OR tribe OR tribal OR reservation OR "American Indian/Alaska Native" OR AI/AN OR AIAN OR "Indian Country" OR "urban Indian")
Embase (Elsevier)*	('randomized controlled trial'/exp OR 'randomized controlled trial')	('american indian'/exp/mj OR 'american indian')
Web of science (Clarivate)*	Topic: ("Randomized Control Trial" OR "Randomized controlled trial" OR RCT)	Topic: ("American Indian" OR "Alaska Native" OR "Native American" OR Indigenous OR tribe OR tribal OR reservation OR "American Indian/Alaska Native" OR AI/AN OR AIAN OR "Indian Country" OR "urban Indian")

Psych Info (EBSCO)*	(DE "Randomized Controlled Trials" OR DE "Randomized Clinical Trials" OR DE "Clinical Trials" OR "Randomized Control Trial" OR "Randomized controlled trial" OR RCT)	(DE "American Indians" OR "American Indian" OR “Alaska Native” OR “Native American” OR Indigenous OR tribe OR tribal OR reservation OR "American Indian/Alaska Native" OR AI/AN OR AIAN OR "Indian Country" OR "urban Indian")
Google Scholar*	("Randomized Control Trial" OR "Randomized controlled trial")	("American Indian" OR “Native American”)
CINAHL (EBSCO)*	(MH "Randomized Controlled Trials") OR (MH "Clinical Trials") OR ("Randomized Control Trial" OR "Randomized controlled trial" OR RCT)	(MH "Native Americans") OR (MH "Medicine, Native American Traditional") OR ("American Indian" OR “Alaska Native” OR “Native American” OR Indigenous OR tribe OR tribal OR reservation OR "American Indian/Alaska Native" OR AI/AN OR AIAN OR "Indian Country" OR "urban Indian")
iPortal Indigenous Studies Portal	Randomized Control Trial	
Native Health Database	"Randomized Controlled Trial" and also separate search with "randomized control trial"	
International Journal of Indigenous Health Database	Manual search	
